# Chitosan-Based Biomaterials for Bone Tissue Engineering Applications: A Short Review

**DOI:** 10.3390/polym14163430

**Published:** 2022-08-22

**Authors:** Antonia Ressler

**Affiliations:** Faculty of Chemical Engineering and Technology, University of Zagreb, Marulićev trg 19, HR-10000 Zagreb, Croatia; aressler@fkit.hr; Tel.: +385-01-4597-237

**Keywords:** biomaterials, bone regeneration, chitosan, composite, polymer, scaffold

## Abstract

Natural bone tissue is composed of calcium-deficient carbonated hydroxyapatite as the inorganic phase and collagen type I as the main organic phase. The biomimetic approach of scaffold development for bone tissue engineering application is focused on mimicking complex bone characteristics. Calcium phosphates are used in numerous studies as bioactive phases to mimic natural bone mineral. In order to mimic the organic phase, synthetic (e.g., poly(ε-caprolactone), polylactic acid, poly(lactide-co-glycolide acid)) and natural (e.g., alginate, chitosan, collagen, gelatin, silk) biodegradable polymers are used. However, as materials obtained from natural sources are accepted better by the human organism, natural polymers have attracted increasing attention. Over the last three decades, chitosan was extensively studied as a natural polymer suitable for biomimetic scaffold development for bone tissue engineering applications. Different types of chitosan-based biomaterials (e.g., molded macroporous, fiber-based, hydrogel, microspheres and 3D-printed) with specific properties for different regenerative applications were developed due to chitosan’s unique properties. This review summarizes the state-of-the-art of biomaterials for bone regeneration and relevant studies on chitosan-based materials and composites.

## 1. Introduction

The incidence of bone disorders has increased, as a result of the aging population coupled with increased obesity and poor physical activity, drawing extensive attention to bone repair medicine research [1,2]. When the bone disorder exceeds the critical size defect (>2 cm), the bone tissue cannot heal by itself and clinical treatment is required [2]. Bone grafting is one of the most common methods for bone regeneration, with over two million bone graft procedures conducted worldwide annually. Numerous types of bone grafts have been used in bone tissue engineering in the last few decades; however, increasing attention is directed towards the biomimetic approach in scaffold design, where molecular, structural and biological compatibility with complex native bone tissue is achieved [3]. When fundamental limitations of biomaterials of first and second generations were recognized, studies shifted to the biomimetic approach and biomaterials that stimulate specific cellular responses at the level of molecular biology [4]. For the successful development of biomimetic scaffolds for bone regeneration, and the role of inorganic and organic phases in the bone tissue, a detailed understanding of the bone composition is essential.

**Figure 1:**
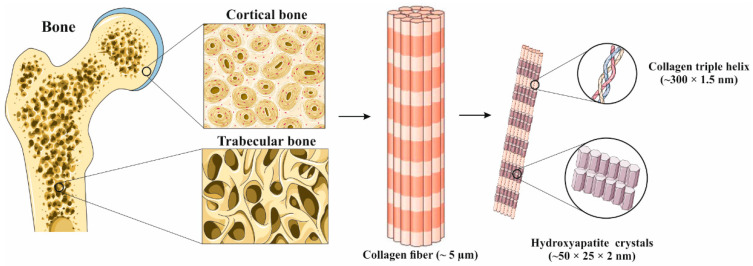
The hierarchical structure of bone at various length scales. Adapted from [12] with permission from Elsevier.

Bone is a heterogeneous composite material consisting of a mineral phase, calcium-deficient carbonated hydroxyapatite (CDHAp, Ca_10−x_(PO_4_)_6−x_(HPO_4_)_x_(OH)_2−x_), and organic phase, consisting of ~90% collagen type I, ~5% non-collagenous proteins, ~2% lipids and water [5,6,7]. The various ionic substitutions (e.g., Mg^2+^, Sr^2+^, Na^+^, K^+^, CO_3_^2−^) in the biological CDHAp structure result in a remarkably complex crystal structure with unique biological properties [7]. Proteins in the bone extracellular matrix can be divided into (i) structural proteins (collagen and fibronectin) and (ii) proteins with specialized functions (e.g., regulation of collagen fibril diameter, signaling molecules, growth factors, enzymes) [8]. Although these proteins are present in the bone structure in a relatively small amount of the total protein mass, they modulate a wide variety of bone key role functions such as regulation of mineralization, cell adhesion and bone resorption/remodeling [6]. Cortical bone is a dense outer surface of bone that forms a protective layer around the inner part, spongy or trabecular bone, in which the main metabolism functions occur [5]. The building blocks of bone tissue are mineralized collagen fiber, composite biomaterial of collagen type I and nano-sized CDHAp. The CDHAp crystals are deposited in parallel with the collagen fibers, and they are later formed by self-assembly of the collagen triple helix [7,9]. Type I collagen is a right-handed helix composed of three left-handed helix polypeptide chains with nonhelical ends, with molecular dimensions of ~300 nm in length and ~1.5 nm in diameter. The collagen triple helix is stabilized via direct inter-chain hydrogen bonds and inter- and intra-chain water-mediated hydrogen bonds [10]. Along with type I collagen, osteocalcin is the next most abundant protein within the bone organic matrix and plays a major role as a structure-directing molecule. It is assumed that osteocalcin mediates the nucleation and growth of platelet-shaped (~50 × 25 × 2 nm) CDHAp crystals [11]. Figure 1 shows a hierarchical structure of typical bone at various length scales. On the macro-length scale, the structure of cortical or compact bone consists of circles in cross-section (Haversian systems) with osteonic canals, while the trabecular part of the bone has a highly porous structure. On the nano-length scale, the structure framework is collagen fibers composed of bundles (triple helix) of mineralized collagen fibers [9].

**Figure 2:**
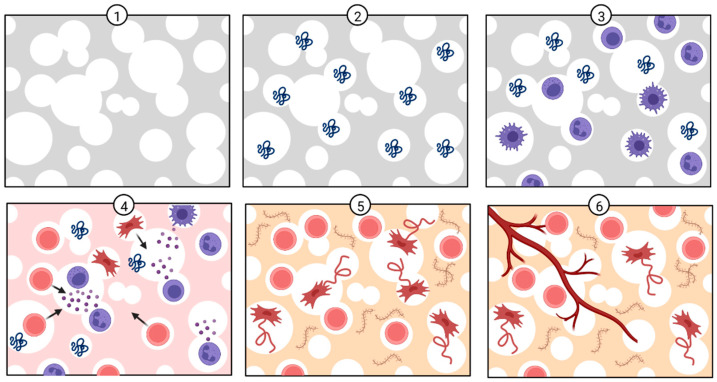
In vivo bone regeneration process after scaffold implantation. Created with BioRender.com (accessed on 30 June 2022).

As biological apatites are characterized by various ionic substitutions that are crucial for bone metabolism, numerous studies have focused on the synthesis and characterization of biomimetic ionic-substituted hydroxyapatite, which is used as a bioactive phase in biomaterials for bone regeneration [12]. As a lot of efforts are put into mimicking the inorganic phase of the bone tissue, the same efforts are directed towards mimicking the organic phase. Combination of these mimicking biomaterials leads to composite material with a complex structure similar to natural bone tissue. Naturally derived polymers (e.g., collagen, gelatin, chitosan, glycosaminoglycans, silk fibrin) have been widely used in a variety of tissue engineering applications, as they can mimic a natural extracellular matrix. As natural polymers are building components of biological tissues, they demonstrate excellent biocompatibility in vivo and present a range of ligands and peptides that facilitate cell adhesion and osteogenic differentiation [3]. One of the most widely studied biopolymers is chitosan, a natural aminopolysaccharide with a unique structure and multidimensional properties suitable for a wide range of applications in biomedicine [13]. Along with bone tissue engineering applications, chitosan has been widely applied in drug delivery and gene therapy because of its excellent biocompatibility and biodegradability under physiological conditions [14]. In addition, the chitosan structure allows chemical and mechanical modifications in order to obtain novel properties, functions and applications [13]. Prior to the development of scaffolds with appropriate regenerative properties, the in vivo regenerative process steps need to be taken into account. After scaffold implantation (1), proteins are absorbed in the scaffold interface (2), followed by infiltration of immune cells (3), the release of chemical signals by immune cells to recruit stem cells (4), microenvironment remodeling (5) and vascularization (6), as schematically shown in Figure 2 [14,15].

Constant progress in bone tissue engineering is ensuring the development of novel functional biomaterials that can solve current challenges in the field. However, more efforts are required to ensure the reproducibility of developed biomaterials and standardization of characterization methods, which could increase the ability to compare biomaterials properties conducted in different studies. Joined efforts and frequent analysis of available literature and design requirements could increase the development of scaffolds with appropriate characteristics. In this short review paper, which is organized into several sections, the author first outlines general knowledge about natural bone tissue and natural polymers, followed by chitosan structure and characteristics. The third section provides a summary of requirements for bone scaffold development (biocompatibility, porosity and pore size distribution, mechanical strength, biodegradation) with a focus on chitosan-based materials. The fourth section provides a summary of different methods for the preparation of chitosan-based biomaterials (molded macroporous, fiber-based, hydrogel, microspheres and 3D-printed scaffolds). The last section provides relevant and recent viewpoints from the literature on the composite scaffolds based on chitosan and calcium phosphates, calcium silicate and bioactive glass. Current trends in the design of chitosan-based scaffolds are highlighted and future perspectives are discussed.

## 2. Chitosan Structure and Characteristics

**Figure 3:**
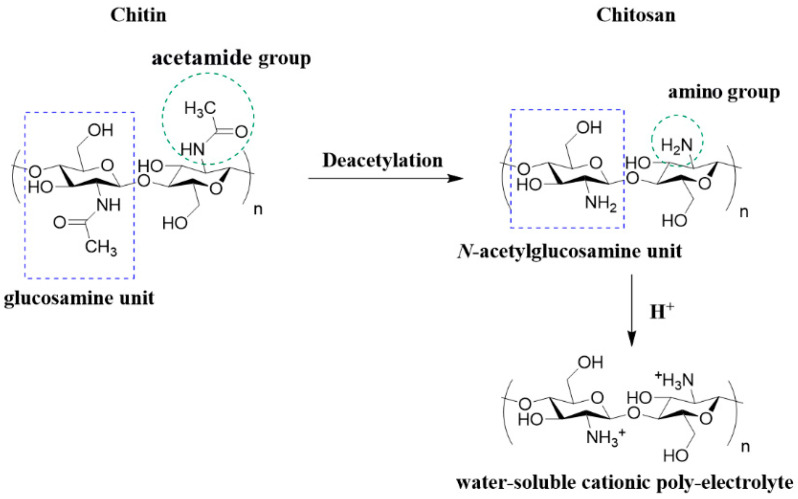
Structure of chitin, chitosan and protonated chitosan (water-soluble poly-electrolyte). The structures were obtained in the ChemDraw 7.0 (PerkinElmer, Waltham, MA, USA) software.

Chitosan is a partially deacetylated derivate of chitin, one of the most abundant polymers in nature found in the shells of crustaceans and walls of fungi. It is composed of randomly distributed β-(1-4)-linked D-glucosamine (glucosamine) and *N*-acetyl-d-glucosamine (*N*-acetylglucosamine) structure units, structurally similar to glycosaminoglycan, a key component of the bone matrix and cell surface which modulates the bioavailability and activity of various osteoclastic and osteogenic factors [5,16,17,18]. Deacetylation of chitin is almost never complete and the chitosan chain still contains amide groups to some extent [16]. The degree of deacetylation (DD, %) is defined as the molar fraction of glucosamine in the chitosan composed of *N*-acetylglucosamine and glucosamine structure units [19]. The DD of chitosan is defined as low (55–70%), middle (70–85%), high (85–95%) or ultrahigh (95–100%), where ultrahigh is difficult to achieve [20]. In Figure 3, the structures of chitin, chitosan and protonated chitosan as a water-soluble poly-electrolyte are shown.

Chitosan has poor solubility in physiological solvents (e.g., water) due to its strong intermolecular hydrogen bonding and it is considered a strong base due to primary amino groups with a pKa value of 6.3 [13,16]. Chitosan solution can be obtained in acidic aqueous (pH < 6) media, which protonate chitosan amino groups, rendering the polymer positively charged and thereby overcoming associative forces between chains [13,16,21]. If the pH of chitosan solution increases above 6, chitosan amino groups become deprotonated and the polymer chain loses its charge, which leads to insolubility. The solubility is highly dependent on the degree of the deacetylation, the used deacetylation method and molecular weight. The solubility of chitosan can be increased by chemical modifications possible at two hydroxyl functional groups in the polymer chain [13]. The detailed review paper by Upadhyaya et al. [22] provides an overview of the water-soluble carboxymethyl chitosan as a modification of the non-soluble chitosan. The highly desired properties of biomaterials designed for applications in the human organism are antibacterial properties without harmful effects on healthy cells. The polycationic nature of the chitosan chain is essential for antibacterial activity. The most probable pathway of chitosan antibacterial activity is by binding to the negatively charged bacterial cell wall (disruption of the cell membrane), followed by attachment to DNA (inhibition of DNA replication) and subsequently cell death. Electrostatic interaction between the polycationic structure and the predominantly anionic components of the microorganisms’ surface, such as Gram-negative lipopolysaccharide and cell surface proteins, plays a key role in antibacterial activity [23].

As previously mentioned, protein adsorption is the first step to take place upon implantation. Protein adsorption occurs within a few minutes or even seconds after scaffold implantation and the cells that reach the biomaterial surface no longer attach directly to the biomaterial but to the adsorbed protein layer. Through cell membrane-bound receptors or ligands, cells identify bioactive binding sites on the protein layer and behave according to the stimuli received [15]. As a natural positive-charged polysaccharide, protonable amino groups on the chitosan backbone electrostatically interact with the various negatively charged proteins [24]. Electrostatic interactions between biomaterial and proteins depend on the biomaterials’ surface and protein charges, which are a function of pH and the solution ionic content. Usually, at low pH, proteins are positively charged, whereas at high pH they are negatively charged [15]. Bovine serum albumin protein is often used as a model protein for biomaterial characterization regarding protein adsorption capacity, because of its high stability, availability at high purity and water solubility [25]. Interactions between BSA protein and chitosan chain depend on the pH and the interaction mechanism is highly complex. BSA protein is negatively charged at neutral pH and the electrostatic interaction of BSA with chitosan is governed by the following two factors: (i) the interaction between protonated chitosan amino groups and the dissociated carboxyl groups of BSA and (ii) the repulsion of the protonated amino groups of chitosan and BSA, as explained by Kim et al. [26]. The protein adsorption capacity of scaffolds needs to be determined, as protein adsorption is the first and crucial step after biomaterial implantation. However, to develop and design a suitable scaffold for bone tissue regeneration, numerous requirements need to be addressed.

## 3. Requirements for Bone Scaffold Development

Scaffolds for bone tissue regeneration must be biocompatible, non-toxic and biodegradable, with an ability to mold into various geometries and forms suitable for cell seeding, migration, growth and differentiation. The structure should mimic the porous and phase structure of the natural bone while maintaining suitable mechanical properties [27,28].

### 3.1. Biocompatibility

Biocompatibility is one of the essential requirements for materials used in tissue engineering applications. Biocompatible materials do not produce a toxic or immunological response in the human body [5]. In almost all published papers, chitosan is described as a non-toxic and biocompatible biopolymer safe for use in the human organism as a scaffold or drug carrier. However, the biocompatibility must be confirmed by biological evaluation for each chitosan-based biomaterial, as they might have different physicochemical characteristics due to different biogenic sources, chitosan type, molecular weight, DD of chitosan and different phases incorporated into the chitosan to obtain composite biomaterials with multifunctional characteristics. In addition, non-cytotoxicity is commonly assessed for 3 or 7 days of cell culture; however, the extended time period of evaluation should be considered. Along with the required extended cell culture time, the appropriate cell lines for bone applications should be used.

### 3.2. Porosity and Pore Size Distribution

Porosity, pore size distribution and pore diameter are some of the most important factors for efficient cell attachment, migration, vascularization and tissue regeneration [29]. Cortical bone has a low porosity of 5–10%, whereas trabecular bone has a porosity of 50–90% [5]. During bone regeneration, interconnected pores in the scaffold are essential for the efficient diffusion of nutrient, oxygen and metabolic waste [30]. In order to design a functional scaffold, along with the porosity in the range of 50–90%, micro- (<20 μm) and macroporosity (>100–400 μm) need to be considered [5]. Microporosity is crucial for cell seeding and retention, capillaries growth, vascularization and cell-matrix interactions. Macroporosity promotes osteogenesis by enhancing cell migration, cell–cell network formation, vascularization, nutrient supply and metabolic waste diffusion [3,30]. Oh et al. [31] systematic study on pore size gradient scaffolds has shown that 380–405 μm pore size has better cell growth for chondrocytes and osteoblasts, whereas the scaffolds with 186–200 μm pore size were better for fibroblasts’ growth. In addition, scaffolds with 290–310 μm pore size showed faster new bone formation than those of other pore sizes. Zhou et al. [32] obtained chitosan-based scaffolds with different bioactive phases, hydroxyapatite and whitlockite, and pore size of ~105 μm. In vivo studies have shown new bone formation within the scaffolds, meaning that pores of ~105 μm meet the requirements for efficient cell seeding and bone ingrowth. An innovative approach to obtain a multi-layered chitosan scaffold with a gradient of pore size (160–275 μm) for osteochondral defect repair was developed by Pitrolino et al. [33]. Osteogenic and chondrogenic differentiation of human mesenchymal stem cells (MSCs) preferentially occurred in selected layers of the scaffold in vitro, driven by the distinct pore gradient and material composition. In the study by Ressler et al. [34], a multi-substituted (Sr^2+^, Mg^2+^, Zn^2+^ and SeO_3_^2−^) calcium phosphate/chitosan composite scaffold with a pore size in the range of 20–350 μm and a porosity of ~75% was prepared by the freeze-gelation method. The requirements for micro- and macroporosity were successfully achieved by adjusting the polymer concentration in the starting solution. Different pore size distributions in these studies indicate that, by using different preparation methods and chitosan concentration of the starting solution, the pore size distribution and porosity can be adjusted and controlled. The pore size distribution and porosity should be some of the main scaffold characteristics considered prior to scaffold development. If the pores are mainly micropores, seeded cells can clog the pores on the scaffold surface and disable diffusion, tissue ingrowth and regeneration. If the pores are mainly macropores, seeding of the cell would not be efficient and that might lead to parts of the scaffold where tissue regeneration is not possible.

### 3.3. Mechanical Strength

The mechanical strength is a critical feature in bone regeneration and it is primarily controlled by pore volume and characteristics of used materials [22]. Optimum balance between porosity, pore size distribution and mechanical properties requirements is still a major challenge in the development of the scaffold. The compressive strength of a trabecular bone is 2–12 MPa, whereas for the cortical bone it is 100–230 MPa [35,36,37]. The mechanical properties of scaffolds for load-bearing applications should be such to successfully replace hard bone tissue [30]. The mechanical characteristics of chitosan scaffolds are significantly lower than the compressive strength and modulus of natural bone tissue. Reported compressive modulus and strength differ depending on the scaffold characteristics, but fall in the ranges of 0.0038–2.56 MPa [38]. Due to poor mechanical properties, chitosan-based scaffolds can be used for non-load-bearing applications, mainly as support for osteoblast cells to adhere, proliferate and differentiate into mature bone cells, producing mineralized extracellular matrix or as drug carriers [39]. Poor mechanical properties limit chitosan-based scaffolds to small bone loss in non-load-bearing implantation areas and improvement of such biomaterials is needed if they would be used for load-bearing applications [39,40]. An innovative approach to improve the mechanical properties of hydroxyapatite/chitosan scaffolds was reported by Rogina et al. [40]. A 3D-printed poly (lactic acid) (PLA) construct was used as a mechanical support, where large pores of 960 ± 50 μm allowed enough space to form a porous composite hydrogel by freeze-gelation technique. PLA and PLA/chitosan scaffolds show similar linear region behavior under loading with a modulus of 32.3 ± 5.4 and 27.3 ± 3.2 MPa, respectively, whereas composite scaffolds based on PLA and hydroxyapatite/chitosan hydrogel possessed lower stiffness with the modulus of 16.4 ± 2.5 MPa [40]. Depending on the application of chitosan-based scaffolds, mechanical properties are one of the main characteristics that should be considered during scaffold design. The design of a scaffold with appropriate porosity, pore diameter and mechanical properties is still a challenge, as these parameters are correlated and their compensation is required.

### 3.4. Biodegradation

The ideal scaffold for bone regeneration should degrade at the same rate as the new tissue formation. If the rate of degradation is higher than the regeneration rate, the scaffold cannot provide support for the host tissue and the regeneration would not be efficient. At physiological conditions, chitosan undergoes physical (e.g., swelling, cracking, dissolution) and chemical (e.g., depolymerization, oxidation, non-enzymatic and enzymatic hydrolysis) degradation. Hydrolytic degradation of the glycosidic bonds between polysaccharide units occurs at a higher rate, making non-enzymatic hydrolytic mechanisms a minor part of chitosan degradation [41]. Chitosan can be enzymatically degraded in vivo by lysozyme, a polycationic protein present in the extracellular matrix in human bone tissue [30]. Lysozyme breaks the chitosan chain by cleaving the glycosidic bonds between polysaccharide units in the polymer. As a result, the molecular weight of the polymer is reduced until eventual solubility and removal of degradation products occur. The degradation products are non-toxic, mainly composed of glucosamine and saccharide, which can then be easily extracted from the body without interference with organs. The degradation rate by each mechanism is related to the degree of crystallinity, which is controlled by the DD, where higher DD results in a lower degradation rate due to closer chain packing and hydrogen bonding [5]. The lysozyme concentration in the extracellular matrix of human tissues can increase up to 1000-fold the amount usually found in serum (0.95–2.45 μm) [42,43,44]. Therefore, it is important to determine the degradation rate of chitosan scaffolds at lower and higher concentrations of lysozyme to examine scaffold stability under physiological conditions.

## 4. Chitosan Three-Dimensional Scaffolds

**Figure 4:**
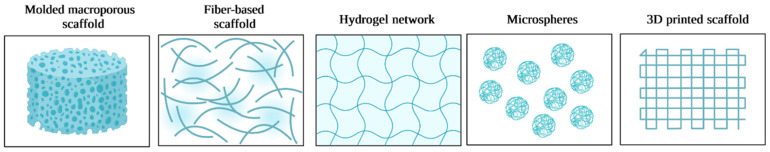
Different designs of chitosan-based three-dimensional scaffolds. Created with BioRender.com (accessed on 30 June 2022).

Due to chitosan’s physical and chemical properties, various types of scaffolds (molded macroporous, fiber-based, hydrogel, microspheres and 3D-printed) can be obtained (Figure 4) for specific treatments that require unique properties. Increasing attention has been gained by 3D-printed chitosan-based scaffolds in recent years, as this technique enables the biofabrication of patient-personalized scaffolds with highly complex geometries. In recent years, a few high-quality review papers on the 3D printing of chitosan, including bioprinting, were published by Rajabi et al. [45], Taghizadeh et al. [46] and Yadav et al. [47].

### 4.1. Molded Macroporous Scaffolds

Compared to the fibers, hydrogel, microspheres and 3D-printed scaffolds, molded chitosan scaffolds are the most studied. The most commonly used method is phase separation and lyophilization, where molded chitosan solution is frozen to allow phase separation [48]. As acetic acid is most commonly used for dissolving chitosan, after the lyophilization the neutralization of chitosan acetate salt is required to prevent scaffold dissolution in the aqueous media. The freeze-gelation method is similar to a previously explained method where after phase separation due to freezing, scaffolds are placed in the gelation solution of sodium hydroxide and ethanol below the chitosan freezing temperature. Following the gelation, scaffolds are washed with ethanol and lyophilized (dried). A combination of the described methods can be used [34,40]. To obtain the desired pore dimension and shape, the polymer concentration, freezing speed and freezing temperature need to be adjusted. In addition, prior to the step phase separation/lyophilization method, porogens can be added to the chitosan solution. The porogens are later leached from the scaffold, leading to additional porosity. When porogens are used without combination with the separation/lyophilization method, the drawback is that this method leads to a lack of control over the interconnectivity of pores inside the scaffold structure. Further, the gas foaming technique can be used alone or in combination with porogens to obtain an open porosity of scaffolds. The high-pressure carbon dioxide (CO_2_) is allowed to saturate the polymeric solution, which causes clusters in the solution and induces porosity [30].

### 4.2. Fiber-Based Scaffolds

Electrospinning is a process that utilizes an electric field to control the deposition of polymer fibers onto target substrates [49]. Compared to synthetic polymers, natural polymers are less spinnable because of limited solubility in most organic solvents, high molecular weight, a polycationic character in solution and three-dimensional networks of strong hydrogen bonds [50]. Fiber-based chitosan scaffolds obtained by electrospinning were highly studied in the 2000s. Homayoni et al. [51] resolved the problem of chitosan high viscosity, which limits its spinnability, through the application of an alkali treatment that hydrolyzes chitosan chains and decreases its molecular weight. Solutions of the treated chitosan in aqueous 70–90% acetic acid produce nanofibers with appropriate quality and processing stability. Optimum nanofibers are achieved with chitosan that is hydrolyzed for 48 h, with a nanofiber diameter of 140 nm. The fiber diameter is strongly affected by the electrospinning conditions and solvent concentration. Recent reviews of the literature for the electrospinning of chitosan-based solutions for tissue engineering and regenerative medicine applications were provided by Qasim et al. [52] and Anisiei et al. [53].

### 4.3. Hydrogel

**Figure 5:**
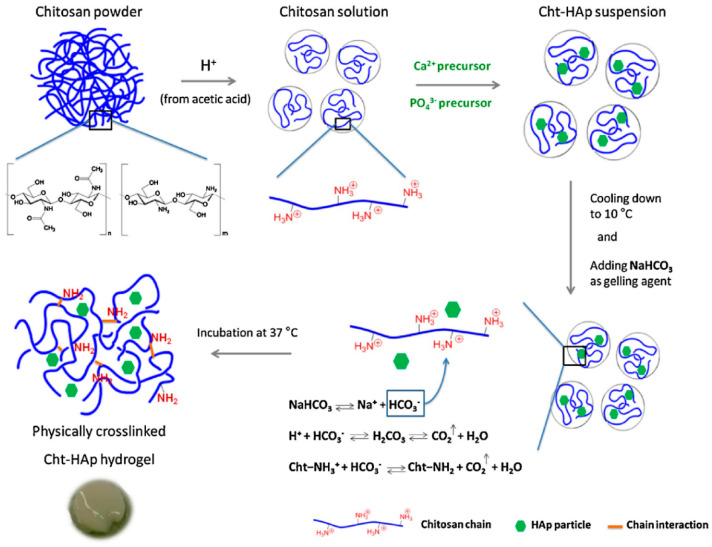
Preparation of physically cross-linked chitosan-hydroxyapatite (Cht-HA) hydrogel, with permission from Elsevier [61].

Conventional methods for applications in tissue engineering (pre-formed hydrogels and scaffolds) face the problem of surgical implantation, increasing the risk of infections and improper scaffold shape and size [54]. In the last decade, smart injectable hydrogels have gained increasing attention because they can be used in minimally invasive treatments [55]. Detailed review papers, regarding mechanisms of injectable hydrogel formation and application in adipose, bone, cartilage, intervertebral discs and muscle tissue engineering were published by Sivashanmugam et al. [54] and Gasperini et al. [56]. Smart injectable gelling systems are liquid at room temperature, and then form gels when injected into the fractured location, filling the complex shape of the defect [55]. Such hydrogels should shorten the surgical operation time, minimize the damage effects of large muscle retraction, reduce the size of scars and lessen post-operative pain, allowing patients to achieve rapid recovery in a cost-effective manner [57]. Hydrogels can be used in non-load-bearing applications to carry and protect cells, proteins, growth factors or drugs, and ensure adequate permeability for the transport of cells’ nutrients and metabolites [58]. A highly important characteristic of injectable hydrogels is gelation time, as slow gelation can cause delocalized gel formation due to the gel precursor diffusion [59]. Hydrogels derived from naturally occurring polysaccharides mimic many features of the extracellular matrix. Therefore, they can direct the migration, growth and organization of encapsulated cells during tissue regeneration [16]. As previously mentioned, chitosan is a pH-responsive polymer, as in mild acids it is soluble and upon neutralization it forms a hydrogel. This occurs due to the removal of repulsive electrostatic interactions during the neutralization process, thereby allowing the amino groups to interact via intermolecular hydrogen bonding [46]. The anionic nature of most human tissues, due to the presence of glycosaminoglycans in the extracellular matrix and the cationic character of chitosan, allows adherence of these hydrogels to tissue sites [19]. In addition, as the cells are negatively charged, positively charged scaffolds are expected to provide a more suitable environment for attachment due to ionic or electrostatic interactions [54]. Due to the polycationic nature of chitosan, pH and thermally induced physical cross-linked hydrogels are highly interesting, as they can be obtained without using cross-linking agents that might be toxic to the human organism. Glycerophosphate salts are widely used for obtaining pH and thermo-sensitive injectable chitosan hydrogels; however, sodium bicarbonate (NaHCO_3_) can also be used as a gelling agent [60,61]. In situ synthesized hydroxyapatite in a chitosan solution (10 °C) was used to obtain pH-responsive hydrogel at 37 °C. A slightly acidic environment of prepared composite solution favors NaHCO_3_ dissociation that releases HCO_3_^−^ ions responsible for carbon dioxide production and pH increases (Figure 5). Although the sol-gel transitions in chitosan solutions with NaHCO_3_ as a gelling agent appeared to be thermally sensitive upon the temperature increase, these systems performed the pH-induced gelation process. The decrease in the apparent charge density of chitosan molecules allows the formation of the three-dimensional chitosan network due to physical junctions of hydrogen bonds [61].

### 4.4. Microspheres

**Figure 6:**
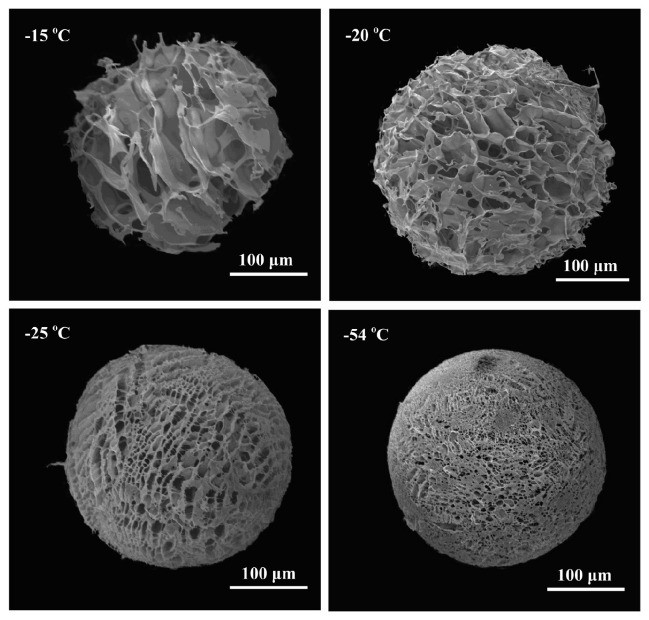
Scanning electron micrographs of porous chitosan microspheres fabricated from chitosan at a concentration of 2% at different temperatures. Reprinted from [64] with permission from Elsevier.

Due to biocompatibility and biodegradability, chitosan microsphere systems have been proposed for use as injectable bone-filling (non-load-bearing) biomaterial and/or drug delivery matrices [62]. Chitosan microspheres for drug delivery and preparation methods were summarized in the review paper by Mitra and Dey [63]. Cell microcarriers in the form of injectable scaffolds offer advantages similar to ones characteristic of injectable hydrogels that repair complex-shaped tissue defects with minimal surgical intervention [64]. Wang et al. [65] fabricated collagen/chitosan-based microspheres (diameter of 200 μm) via the emulsification method by using glutaraldehyde as a cross-linking agent. Obtained microspheres have shown stability for at least 90 days and good biological properties by supporting attachment and proliferation of the cells. As previously described, chitosan degradation products are not toxic for cells and the human organism; however, to obtain stable chitosan microspheres, chemical cross-linking is required to cross-link amino groups in the chitosan chain. As suggested by Fang et al. [64], residual cross-linking agents in microspheres might have a toxic effect towards cells, surrounding tissue and the human organism. Complete removal of unreacted cross-linking agents from obtained scaffolds remains a challenge. To overcome these drawbacks of chemically cross-linked chitosan, the introduction of a bioactive polyanionic biopolymer to interact electrostatically with the amino groups of chitosan to form polyelectrolyte complexes (PEC) has been proposed. Fang et al. [64] obtained poly(l-glutamic acid)/chitosan PEC porous microspheres by electrostatic interactions. It was determined that the pore size distribution, porosity, structure and stability of microspheres are dependent on freezing temperature and polymer concentration (Figure 6). An additional study with an approach free of toxic cross-linking agents was conducted by Huang et al. [66]. Highly porous chitosan microspheres were prepared through an emulsion-based thermally induced phase method with an average diameter of microspheres of ~150 μm and with interconnected pores in the range of 20–50 μm. Obtained microspheres showed excellent biocompatibility with multidirectional cell–cell interactions. Another approach to avoid cross-linking agents to produce stable chitosan-based microspheres is through physical cross-linking via chelation interactions of copper and zinc with chitosan, as recently reported by Lončarević et al. [67] and Rogina et al. [68]. The studies highlight the alternative approach to produce stable chitosan-based microspheres by using simple complexation reactions through transition metal ions.

For usage as a bone-filling biomaterial, the drawbacks of pure chitosan microspheres are lack of bone-binding ability and burst release problems. To overcome limitations, Ding et al. [62] proposed that obtaining composite microspheres based on chitosan and hydroxyapatite can lead to an increase in bone-binding ability. In vivo studies on apatite-coated chitosan microsphere conducted by Xu et al. [69] showed bone formation after 7 days. Further, hydroxyapatite/sodium alginate/chitosan composite microspheres, reported by Bi et al. [70], were prepared by an emulsion cross-link technique where calcium ions were used as a cross-linking agent. However, the microspheres as microcarriers often only enabled cell attachment and growth on the surface due to low or closed porosity [62,66]. Although multiple chitosan-based microspheres have been developed, minority studies report highly porous microspheres with open porosity that enable cell migration and cell–cell interactions in the 3D environment. However, even with low or closed porosity, the advantage of microspheres for use in biomedical applications over a granular approach for bone repair is a larger specific surface area, which can improve cell adhesion and proliferation [70]. In addition, chitosan microspheres can be used as a filler component in molded scaffolds based on other polymers [71], or microspheres can be molded to obtain highly porous scaffolds [72].

## 5. Chitosan Composite Scaffolds

**Figure 7:**
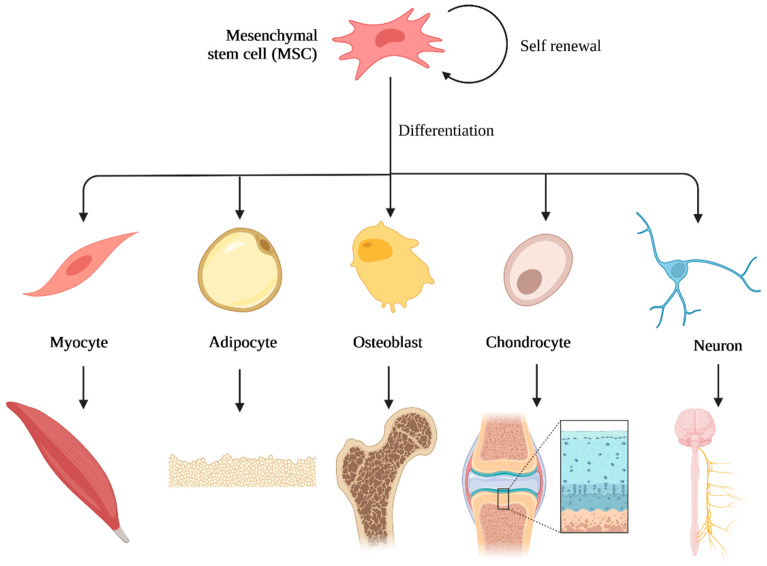
Mesenchymal stem cells differentiation. Created with BioRender.com (accessed on 2 July 2022).

The extracellular matrix in natural bone tissue supports cell attachment, proliferation and differentiation. Scaffolds for bone regeneration should mimic the natural ECM as much as possible. In particular, integration of multiple stimuli in scaffolds including physical (e.g., porosity, pore size distribution, topography, stiffness) and biochemical (e.g., growth factors, key role elements, genes, proteins) factors similar to natural bone tissue will improve scaffold efficacy [3,12]. MSCs are used in regenerative medicine because of their potential for self-renewal and multipotency. Multipotent stem cells can differentiate into multiple lineages (e.g., myocyte, adipocyte, osteoblast, chondrocyte, neuron), as schematically shown in Figure 7 [73,74]. Biomaterials can direct MSC attachment, proliferation and differentiation into different cell types and this can be controlled by the optimization of material characteristics such as composition, geometry, pore size, porosity, topography, stiffness, etc. [75]. Chitosan and its polymer-based composites are often used for the development of materials due to the similarity of the polysaccharide structure to the glycosaminoglycans of cartilage and as it can direct MSCs differentiation towards chondrocyte cell type (chondrogenesis) [76,77,78]. However, in order for chitosan-based scaffolds to mimic natural bone tissue and stimulate differentiation into osteoblast cell types (osteogenesis), chitosan is often combined with inorganic phases. Calcium phosphates, calcium silicates and bioactive glass are among the most studied bioactive components within the chitosan matrix to mimic naturally occurring mineral phases and stimulate the osteogenic differentiation of cells. An innovative approach was reported by Pitrolino et al. [33] and Erickson et al. [79], who obtained a multi-layered chitosan scaffold for osteochondral defect repair with the incorporation of the highly porous layer based on chitosan and hydroxyapatite for bone regeneration.

### 5.1. Calcium Phosphates

Calcium phosphates (e.g., hydroxyapatite, α-tricalcium phosphate, β-tricalcium phosphate) are among the most studied bioactive phases combined with the chitosan matrix. Inspired by natural bone tissue, numerous studies are focused on the scaffolds, where chitosan provides an organic matrix mimicking naturally occurring collagen, while calcium phosphate crystals mimic naturally occurring minerals (apatite). Numerous studies have confirmed the osteogenic properties of chitosan/calcium phosphate-based scaffolds. Rogina et al. [80,81] confirmed that compressive strength and swelling capacity measured in physiological conditions have shown that the critical hydroxyapatite portion, which improves chitosan properties, does not exceed 30 wt%, while further studies in perfusion conditions confirmed the best influence of hydroxyapatite on hMSC proliferation and osteoinduction on composite scaffolds with 30% of hydroxyapatite. A higher apatite fraction indicated poor mineralization of hMSCs extracellular matrix. Siddiqui et al. [82] prepared composite scaffolds based on chitosan and β-tricalcium phosphate cross-linked with genipin, confirming osteogenic differentiation during 21 days of cell culture. In recent years, to mimic the chemical composition of natural apatite, calcium phosphates were substituted with various key role ions and combined with chitosan. Ran et al. [83] obtained Mg-, Zn-, Sr- and Si-doped hydroxyapatite/chitosan hydrogels and confirmed that the Sr-chitosan/hydroxyapatite hydrogel exhibited the highest proliferation potential among the cultured cells compared to the samples with other ions. To further enhance osteogenic properties and improve mechanical properties, Zhang et al. [84] obtained composite scaffolds based on silk fibroin, carboxymethyl chitosan, strontium substituted hydroxyapatite and cellulose. Mansour et al. [85] prepared a chitosan-based scaffold loaded with Ag/Mg-co-substituted hydroxyapatite. Ghorbani et al. [86] prepared an electrospun scaffold (fiber diameter 210 nm) based on PCL, chitosan and zinc-doped hydroxyapatite, showing a positive effect on cell attachment and proliferation. Further, the synergic effect of Sr^2+^, Mg^2+^, Zn^2+^ and SeO_3_^2−^ ions was confirmed in perfusion conditions. It has been determined that ions have a significant influence on the expression of characteristic bone genes (alkaline phosphatase, bone sialoprotein, collagen type I and dentin matrix protein I), phosphate deposits and newly formed bone tissue. Recent studies have shown better osteogenic properties of substituted calcium phosphate/chitosan scaffolds compared to the non-substituted scaffolds [34]. Therefore, further studies can be focused on using substituted calcium phosphates with chitosan and other polymers, such as collagen, which can further increase osteogenic properties.

### 5.2. Calcium Silicate

Along with calcium phosphates and silicates, calcium silicates are promising biocompatible ceramic materials that can provide a microenvironment suitable for bone tissue regeneration. Along with Ca^2+^ ions, silicate ions have a key role in the bone regeneration process, as they can regulate MEK and PKC pathways [87]. In a recent study by Zhou et al. [88], it has been demonstrated that calcium silicate had significantly greater osteoinductive capacity both in vitro and in vivo compared with the traditional clinically used β-tricalcium phosphate bioceramics. Further, ionic substitutions are not only studied for calcium phosphates. In recent years, increasing attention has been directed towards substituted calcium silicates, especially for substitutions with Sr^2+^ ions [89,90,91]. Due to the positive effect of calcium silicates on bone regeneration, significant efforts have been directed toward obtaining scaffolds based on chitosan and calcium silicates. Peng et al. developed a lanthanum- [87] and gadolinium [92] -doped mesoporous calcium silicate/chitosan scaffold, which supports the adhesion, proliferation and differentiation of MSCs. Lin et al. [93] enhanced calcium silicate properties by obtaining composite scaffolds based on chitosan to ensure the antibacterial properties of the scaffolds. Mukherjee et al. [94] reported improved osteoblast function (viability, adhesion and proliferation) on titanium implant surfaces coated with a nanocomposite based on apatite, wollastonite (CaSiO_3_) and chitosan. A significant increase in the expression of osteocalcin and mineralization, compared to a non-treated substrate, confirmed the biocompatibility of the composite coating and its ability to initiate early osseointegration. Further, Genasan et al. [95] confirmed that the addition of calcium silicate (40% *w*/*w*) into gellan-chitosan scaffolds induces osteogenic differentiation of mesenchymal stromal cells where significant depositions of minerals, along with the expression of osteogenic genes, including BMP2, Run2, osteocalcin and osteonectin, were detected. Even though the calcium silicate-based scaffolds have shown desired properties for bone regeneration, more studies are focused on scaffold development and characterization based on calcium phosphates. Future studies should be focused on the development of scaffolds based on calcium silicates and substituted calcium silicates within the chitosan matrix and should be compared to similar scaffolds based on calcium phosphates and chitosan.

### 5.3. Bioactive Glass

Along with calcium phosphates, bioactive glasses are used as bioactive fillers in chitosan-based scaffolds to increase cell response and osteogenic properties. Bioactive glasses are widely used for bone tissue regeneration due to their chemical interactions in vivo, where osteointegration is promoted by the formation of a calcium phosphate layer [96]. When included in the chitosan matrix, bioactive glasses enhance the metabolic activity of cells and mineralization [97]. Saatchi et al. [98] reported chitosan/polyethylene oxide nanofibrous scaffolds containing different amounts of cerium-doped bioactive glass. It has been determined that increasing the content of cerium-doped bioactive glass, cell adhesion and spreading have been enhanced. Further, fibroblast cells spread across the composite scaffold and took a 3D shape; however, there was no sign of cell expansion on the polymer scaffold without cerium-doped bioactive glass. In addition, composites based on chitosan and bioactive glasses are used as coating materials on AZ91 Mg alloy [99], 316 L stainless steel [100], WE43 Mg alloy [101,102] and Ti-6Al-4V [103] to improve the biocompatibility and bioactivity of metallic substrates for biomedical applications. To prevent the formation of biofilm on orthopedic implants, coatings based on chitosan and bioactive glasses can be combined with different drugs (e.g., vancomycin) to prevent the adhesion and proliferation of bacteria, as reported by Zarghami et al. [104]. Further, Sergi et al. [105] prepared a composite based on commercial passive gauzes, chitosan and bioactive glass doped with Sr^2+^, Mg^2+^ and Zn^2+^ ions for wound healing. It was determined that wound dressings with obtained composite material showed higher bioactivity compared to wound dressings with pure chitosan. The release of Sr^2+^, Mg^2+^ and Zn^2+^ ions enhanced cell proliferation and wound healing rate. A composite system based on chitosan and doped bioactive glass could be further examined for bone tissue engineering applications. In addition to a research paper, a detailed review paper by Sergi et al. [106] provided an overview of studies on bioactive glasses and natural polymer composites for medical devices for both soft and hard tissues. Due to different ions present in the bioactive glasses, further studies should provide additional comparative studies and a better understanding of the significance of each ionic component in bioactive glasses and its influence on the osteogenic properties of chitosan scaffolds.

## 6. Conclusions and Future Perspective

Various chitosan-based materials have been designed and reported in the literature. However, more efforts are required to address current challenges to bring developed biomaterials to clinical use and application. Prior to the scaffold design, the researcher should consider all requirements for in vivo studies, clinical trials and mass productions. *ISO 10993 Biological evaluation of medical devices—Part 1: Evaluation and testing within a risk management process* should be considered prior to biological evaluation. Further, the characterization of chitosan-based materials for bone regeneration is not standardized. Even if there is a large number of papers on chitosan-based scaffolds as potential materials for bone regeneration, different methods for characterization are used. Therefore, the results of different studies cannot be properly compared and a final conclusion on material potential cannot be conducted. In recent years, the standardization of protocols and regulation of biomaterials has become highly required in order to improve technology transfer and increase the amount of commercially available products [107,108,109]. An additional challenge characteristic for naturally derived polymers is that the different properties depend on the source and preparation method. This further disables comparison between obtained scaffolds from different studies. With joint efforts from researchers by following requirements for biomaterials design and characterization, more developed biomaterials could be translated to clinical trials and be approved for commercial use.
